# Reaction Mechanism of Aluminum Toxicity on Leaf Growth of Shatian Pomelo Seedlings

**DOI:** 10.3390/plants14040603

**Published:** 2025-02-17

**Authors:** Dan Tan, Jingfu Yan, Yali Yang, Shaoxia Yang, Lubin Zhang, Yingbin Xue, Ying Liu

**Affiliations:** 1 Department of Agronomy, College of Coastal Agricultural Sciences, Guangdong Ocean University, Zhanjiang 524088, China; 2 Guangdong Provincial Key Laboratory of Conservation and Precision Utilization of Characteristic Agricultural Resources in Mountainous Areas, Jiaying University, Meizhou 514015, China

**Keywords:** *Citrus maxima “Shatian Yu”*, Al toxicity stress, leaves, regulatory mechanisms

## Abstract

This study aimed to examine the effects of aluminum (Al) stress on the leaves of Shatian pomelo (*Citrus maxima “Shatian Yu”*) and its underlying response mechanisms. Leaf phenotype analysis, physiological response index determination, transcriptome analysis, and genome verification were employed to investigate the effects of Al toxicity in detail. Al toxicity stress inhibited leaf growth and development, reducing leaf area, girth, and both dry and fresh weights. Antioxidant enzyme activity and soluble protein content in leaves significantly increased with rising Al stress levels. Additionally, Al toxicity caused an accumulation of Al ions in leaves and a decline in boron, magnesium, calcium, manganese, and iron ion content. RNA sequencing identified 4868 differentially expressed genes (DEGs) under 0 mM (Control) and 4 mM (Al stress) conditions, with 1994 genes upregulated and 2874 downregulated, indicating a complex molecular regulatory response. These findings were further validated by real-time quantitative PCR (qPCR). The results provide critical insights into the molecular mechanisms of Shatian pomelo leaf response to Al toxicity and offer a theoretical basis and practical guidance for improving citrus productivity in acidic soils.

## 1. Introduction

Soil acidification represents a critical environmental challenge that adversely impacts agricultural productivity and plant health globally [1]. In acidic soils, the availability of heavy metals, particularly aluminum (Al), increases significantly, posing a serious threat to plant growth and development [2]. Al is one of the most prevalent metallic elements in the Earth’s crust [3]; however, under acidic conditions, it can exist in a free form, leading to toxic effects on plant roots and leaves [4]. The presence of Al toxicity in acidic soils severely restricts crop yield and quality [5].

*Citrus* spp., as an important cash crop in the world, is widely planted in tropical and subtropical areas, and its growing environment is mostly acidic soil, so it is particularly vulnerable to the influence of Al poisoning [6]. Studies have shown that Al stress can inhibit the root growth of citrus plants, affect the photosynthetic efficiency, interfere with the nutrient absorption of plants, and ultimately lead to plant growth retardation, yield reduction, and fruit quality reduction [7]. In addition, Al stress can also induce an increase in the level of reactive oxygen species (ROS) in plants, leading to oxidative stress and, thus, damaging the structure and function of cell membranes [7]. In order to cope with Al toxicity, citrus plants have evolved a series of adaptive mechanisms, including the activation of antioxidant defense systems [6].

The Shatin pomelo *(Citrus maxima* “Shatian Yu”) is a widely recognized citrus fruit native to Southeast Asia, particularly flourishing in tropical and subtropical regions [8]. Citrus plants are predominantly evergreen fruit trees found in these areas, typically thriving in acidic soils, which make them susceptible to Al toxicity [9,10]. Elevated levels of Al can severely inhibit various growth parameters, including bud length, leaf count, leaf area, fresh bud weight, and chlorophyll content in plant seedlings; this also disrupts the effective rate of photosynthesis, significantly impacting overall plant growth [11]. Research has identified that Al stress negatively affects the reaction center of the photosystem I (PSI) in rhododendron (*Rhododendron simsii*) [12]. For instance, as Al concentration escalates from 0 mM to 8 mM, the circumference, leaf area, and both dry and fresh weight of peanut leaves diminish significantly by 49.99%, 39.38%, 48.11%, and 56.80%, respectively [13]. Under Al stress, the epicotyl length and weight of mung bean (Phaseolus radiatus) are inhibited, resulting in decreases in plant height and total biomass [14]. Moreover, Al stress hampers the accumulation of essential nutrients in plants and reduces leaf area in mung beans [14]. At the cellular level, excessive Al exposure compromises the integrity of cell walls, plasma membranes, cytoskeletons, and nuclei [15]. Additionally, the levels of *monogalactosyldiacylglycerol* (*MGDG*) and *digalactosyldiacylglycerol* (*DGDG*) in *Arabidopsis thaliana* decrease significantly under Al stress, thus affecting both the stability and permeability of the plasma membrane [16].

Al stress also leads to an imbalance of reactive oxygen species (ROS) [17]. For example, the activity of superoxide dismutase (SOD) in the leaves of tea trees (*Camellia sinensis*) markedly increases with rising Al concentrations [18]. Additionally, under 100 μM Al stress, the levels of malondialdehyde (MDA), hydrogen peroxide (H_2_O_2_), and proline (PRO) in broad bean (Vicia faba) leaves rise, indicating that Al stress induces oxidative stress in these plants [19]. Furthermore, the activity of ascorbate peroxidase (APX) in spinach (*Spinacia oleracea*) is elevated under Al stress [20].

RNA sequencing (RNA-Seq) has become an increasingly prevalent technique for investigating gene expression profiles [21]. Research on Al-stressed plants has led to the identification of gene modifications associated with membrane transporters, signal transduction, transcription factors, oxidative stress, cytoskeletal dynamics, energy, and metabolism [22]. For instance, a study utilizing RNA-Seq and real-time fluorescence quantitative PCR (qPCR) analysis in tomato (*Solanum lycopersicum*) revealed the presence of 53 SlAAEs (acyl-activating enzymes, AAEs) in tomato root tips [23]. Under Al stress, nine of these genes exhibited significant changes, with eight differentially expressed genes (DEGs) being upregulated and one DEG downregulated [23]. Despite these insights, the molecular regulatory mechanisms governing the response of Shatian pomelo leaves to Al toxicity remain poorly understood.

As a common environmental stress factor, Al stress has been proven to have significant negative effects on the growth and development of many kinds of plants [4,5,9]. In acidic soil, the activity of Al ions increases significantly, which makes it easy to exist in a free state and be absorbed by plants, thus causing a toxic effect on plant leaves [4,5,9,10]. In this study, it was hypothesized that Al stress could not only inhibit the growth of Shatian pomelo leaves but also significantly reduce the dry weight, fresh weight, leaf area, and perimeter of leaves and also significantly affect the antioxidant enzyme activity, soluble protein content, and the absorption of various nutrient elements in leaves. Specifically, Al stress might lead to a significant increase in the activities of antioxidant enzymes (such as APX, CAT, POD, and SOD) in leaves in response to oxidative stress. At the same time, soluble protein content might be increased to maintain osmotic pressure and structural stability of cells. In addition, Al stress may lead to a significant decrease in the content of boron (B), calcium (Ca), magnesium (Mg), manganese (Mn), iron (Fe), and other nutrient elements, thus affecting the normal physiological function of plants. The aim of this study is to verify the correctness of these hypotheses through detailed physiological and molecular analysis, so as to provide a theoretical basis for understanding the growth restriction mechanism of Shatian pomelo in acidic soil and to provide scientific guidance for the improvement of acidic soil and cultivation of Al-resistant varieties in agricultural practice. This study employs high-throughput deep sequencing technology to analyze the transcriptome of Shatian pomelo leaves under Al toxicity stress, further investigating the effect of Al on these leaves and its associated tolerance mechanism. The findings will provide essential theoretical insights and practical guidance for enhancing the productivity of citrus plants in acidic soils.

## 2. Results

### 2.1. Effects of Al Poisoning on Leaf Phenotypic Indexes of Shatian Pomelo Plants

**Figure 1:**
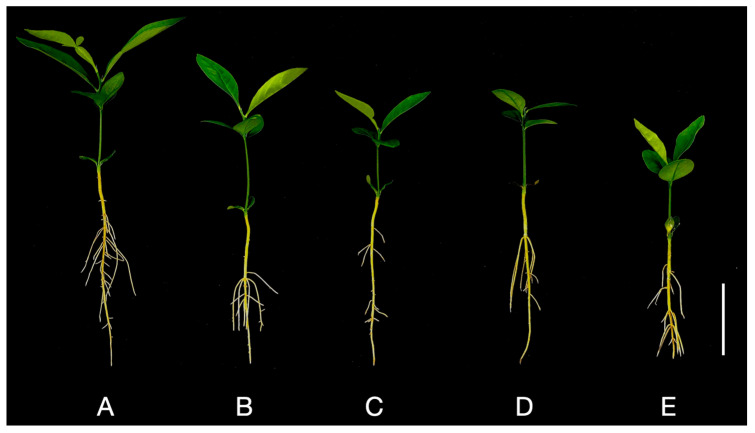
Phenotypes of leaves from the Shatian pomelo plants after 20 days treated with different Al concentrations: (**A**) 0, (**B**) 1, (**C**) 2, (**D**) 4, and (**E**) 8 mmol/L Al (bar = 5 cm).

**Figure 2:**
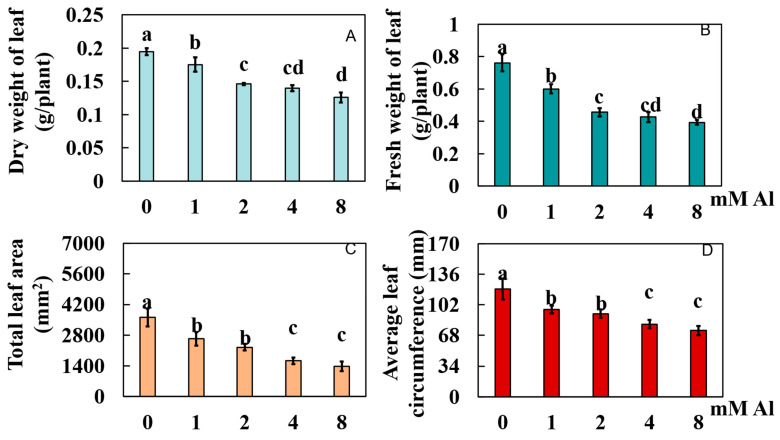
Effects of different Al concentrations on leaf growth index of Shatian pomelo. The control group was treated at 0 mmol/L Al, while the experimental group was treated at 1.0, 2.0, 4.0, and 8.0 mmol/L Al, respectively. After 20 days, Shatian pomelo leaves were collected for data measurement: (**A**) dry leaf weight, (**B**) fresh leaf weight, (**C**) leaf area, and (**D**) leaf circumference. The results were expressed as the mean and standard deviation of three repeated biological experiments. Waller–Duncan multiple comparison tests and one-way analysis of variance were used to compare the significant differences between the control group and the treatment group. Different letters on the bar chart indicated significant differences between the data (*p* < 0.05).

The phenotypic indices of pomelo leaves subjected to various Al concentrations were assessed (Figure 1A–E and Figure 2A–D). When compared to the control group (0 mmol/L Al), the dry weight of leaves in the treatment groups (1, 2, 4, and 8 mmol/L Al) decreased significantly by 9.87%, 24.92%, 28.16%, and 35.35%, respectively (Figure 2A). Leaf fresh weight was also significantly reduced by 21.18%, 40.15%, 43.98%, and 48.31%, respectively (Figure 2B). The blade area showed a significant decrease of 27.12%, 37.71%, 54.70%, and 61.56%, respectively (Figure 2C). Additionally, the leaf circumference fractions decreased significantly by 19.15%, 23.12%, 32.78%, and 38.76%, respectively (Figure 2D).

### 2.2. Effects of Al Poisoning on Physiological Response Indexes of Shatian Pomelo Leaves

**Figure 3:**
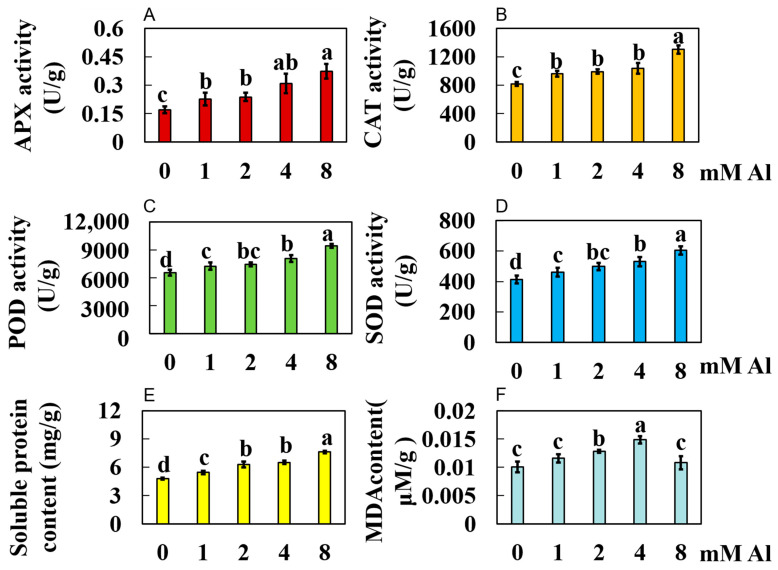
Results of physiological response indexes of Shatian pomelo leaves under Al toxicity stress for 20 days. The leaves of Shatian pomelo were collected and 8 physiological indexes were determined: (**A**) APX (ascorbate peroxidase) activity, (**B**) CAT (catalase) activity, (**C**) POD (peroxidase) activity, (**D**) SOD (superoxide dismutase) activity, (**E**) soluble protein content, and (**F**) MDA (malondialdehyde) content. The results were expressed as the mean and standard deviation of three repeated biological experiments. Waller–Duncan multiple comparison tests and one-way analysis of variance were used to compare the significant differences between the control group and the treatment group. Different letters on the bar chart indicated significant differences between the data (*p* < 0.05).

In comparison to the control group (0 mmol/L Al), as the Al concentration increased in the treatment groups (1, 2, 4, and 8 mmol/L Al), APX activity significantly increased by 33.35%, 39.49%, 81.07%, and 119.04%, respectively (Figure 3A). Catalase (CAT) activity exhibited significant increases of 17.56%, 21.12%, 26.70%, and 59.28%, respectively (Figure 3B). Peroxidase (POD) activity rose significantly by 10.47%, 13.67%, 23.10%, and 43.83%, respectively (Figure 3C). SOD activities increased significantly by 11.65%, 20.87%, 28.64%, and 46.37%, respectively (Figure 3D). The content of soluble proteins increased significantly by 13.09%, 30.97%, 35.34%, and 58.42%, respectively (Figure 3E). Finally, MDA content rose by 14.75%, 26.82%, 47.01%, and 7.05%, respectively (Figure 3F).

### 2.3. Influence of Al Poisoning on Accumulation of Several Elements in Shatian Pomelo Leaves

**Figure 4:**
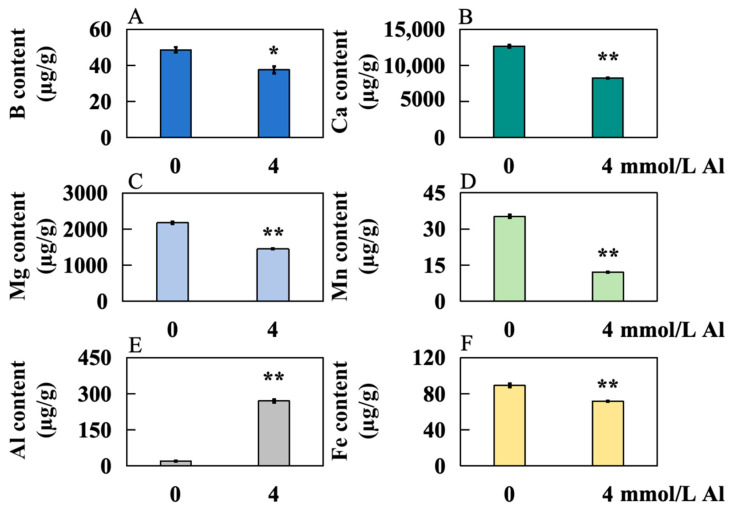
Accumulation of 6 elements in Shatian pomelo leaves after Al poisoning treatment for 20 days. The concentrations of 6 elements, (**A**) boron (B), (**B**) calcium (Ca), (**C**) magnesium (Mg), (**D**) manganese (Mn), (**E**) Al, and (**F**) iron (Fe), were determined. The results were expressed as the mean and standard deviation of three repeated biological experiments. Student’s *t*-test was used to test the significance of the difference between the control group and the Al poison treatment group. An asterisk on the bar chart indicated a significant difference (* *p* < 0.05) or a very significant difference (** *p* < 0.01) between the data.

The concentrations of boron (B), calcium (Ca), magnesium (Mg), manganese (Mn), Al, and Fe in Shatin pomelo leaves were measured after exposure to 0 (control group) and 4 mmol/L Al (Al toxicity stress) for a duration of 20 days. Compared to the control group, the levels of B, Ca, Mg, Mn, and Fe significantly decreased by 22.78%, 34.70%, 33.27%, 65.65%, and 19.49%, respectively (Figure 4B–D,F). In contrast, the Al content experienced a significant increase of 1205.80% (Figure 4F).

### 2.4. RNA-Seq Analysis of Shatian Pomelo Leaves

**Figure 5:**
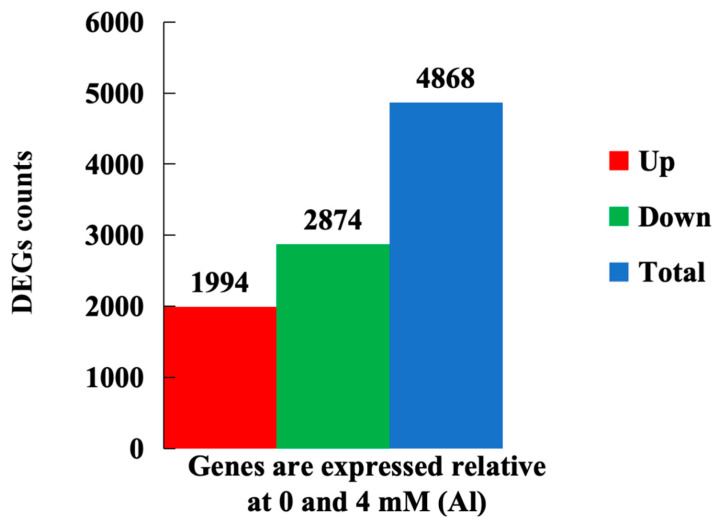
Statistical histogram of DEGs. Red represented the number of upregulated genes, green represented the number of downregulated genes, and blue represented the total number of DEGs.

Transcriptome sequencing analysis of Shatian pomelo leaves subjected to 0 (control group) and 4 mmol/L Al (Al toxicity stress) for 20 days revealed a total of 4868 DEGs under Al toxicity stress, among which 1994 genes were upregulated and 2874 genes were downregulated (Figure 5 and Tables S1–S3).

### 2.5. Functional Enrichment Analysis of DEGs

**Figure 6:**
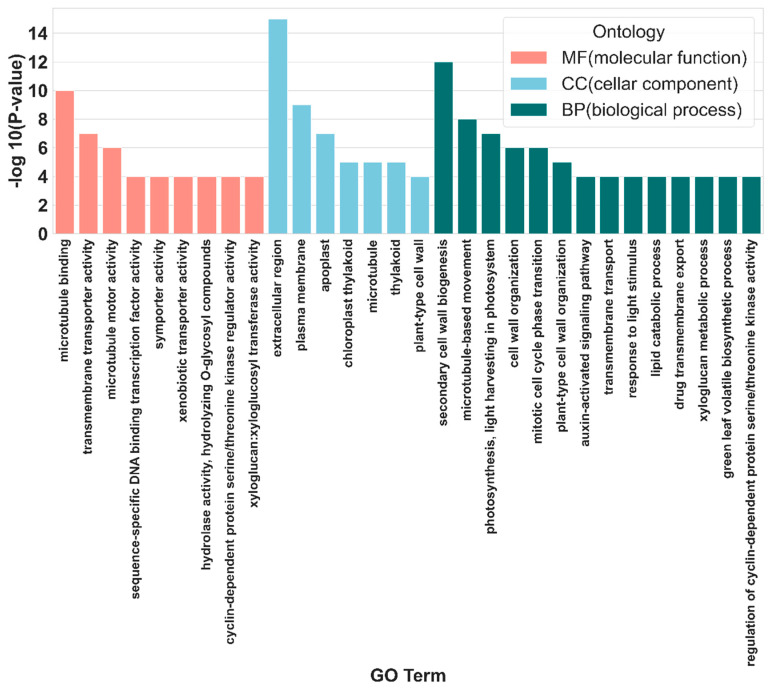
Histogram of GO (gene ontology) enrichment *p* value. The horizontal coordinate was the enriched GO term, and the vertical coordinate was the term-log10 (*p*-value) value.

Functional enrichment analysis indicated that the DEGs in Shatin pomelo leaves were primarily associated with cellular composition (CC), followed by biological processes (BP) and molecular functions (MF) (Figure 6 and Table S4).

**Figure 7:**
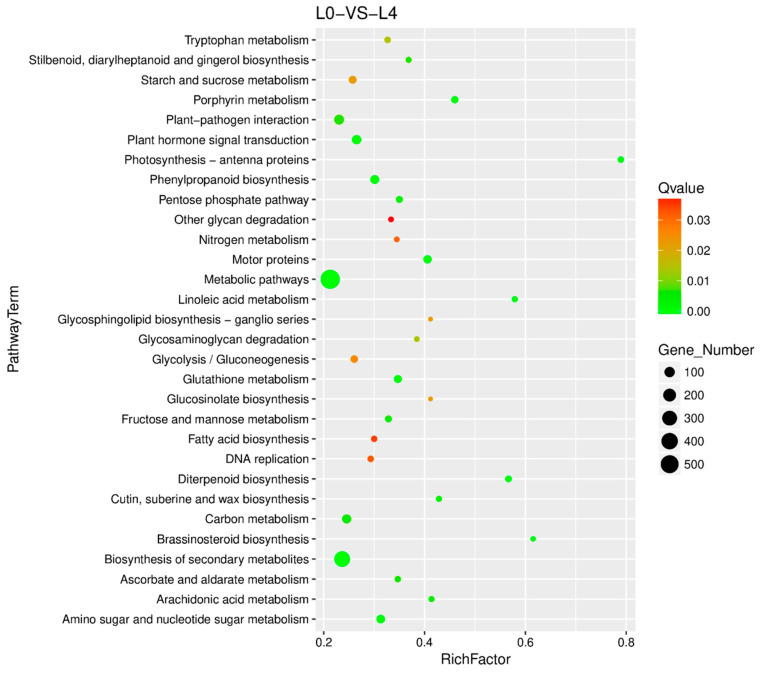
Bubble diagram of KEGG enrichment. The vertical axis represented the pathway name, the horizontal axis represented the rich factor, the size of the dots represented the number of DEGs in this pathway, and the color of the bubbles corresponded to different Q value ranges. The 30 pathway entries with the most significant enrichment were selected to be displayed in this figure. If there are less than 30 enriched pathway entries, all of them will be displayed. KEGG enrichment was assessed by enrichment factor (RF), false positive rate (FDR), and the number of genes clustered in the pathway. RF referred to the number of DEGs in pathway entries as a percentage of the total number of genes in pathway entries in all interpreted genes. The higher the RF, the higher the aggregation level. Generally, the range of FDR was 0–1, and the closer to zero, the more significant the enrichment.

In the leaves of Shatian pomelo, KEGG enrichment was prominently observed in the following pathways: metabolic pathways (591 genes; comprising 265 upregulated and 326 downregulated genes), biosynthesis of secondary metabolites (341 genes, with 180 upregulated and 161 downregulated genes), and plant–pathogen interactions (88 genes, consisting of 52 upregulated and 36 downregulated genes) (Figure 7 and Table S5). Additionally, pathways related to plant hormone signaling (66 genes, including 25 upregulated and 41 downregulated genes), carbon metabolism (66 genes, comprising 49 upregulated and 17 downregulated genes), amino sugar and nucleotide sugar metabolism (46 genes, including 19 upregulated and 27 downregulated genes), motor proteins (41 genes, with no upregulated genes and 41 downregulated genes), and starch and sucrose metabolism (35 genes, including 17 upregulated and 18 downregulated genes) were also identified (Figure 7 and Table S5).

### 2.6. Identification of Hormone-Related Genes

A total of 60 DEGs associated with hormone synthesis were identified in the leaves of Shatian pomelo under Al toxicity stress (4 mmol/L Al) (Table S6). Among these, 40 genes were related to auxin synthesis, with 7 genes upregulated and 33 genes downregulated. Two genes involved in abscisic acid (ABA) synthesis were downregulated (*Cg5g010670* and *Cg5g001590*), whereas one gene was upregulated (*Cg8g018300*). Additionally, 8 genes related to gibberellin synthesis were downregulated, whereas 9 genes were upregulated.

### 2.7. Identification of DEGs of Antioxidant Enzymes

Thirty-one DEGs related to antioxidant enzymes were identified in the leaves of Shatian pomelo under Al toxicity stress (4 mmol/L Al) (Table S7). Among these DEGs, 18 are associated with peroxidase; 12 of these genes (*Cg3g024510*, *Cg2g042930*, *Cg7g014170*, *Cg5g004520*, *Cg1g004840*, *Cg6g023390*, *Cg1g001240*, *Cg2g006540*, *Cg2g018020*, *Cg1g008440*, *Cg3g018770*, and *Cg2g001400*) were downregulated, whereas 6 genes (*Cg2g001370*, *Cg8g005280*, *Cg1g009400*, *Cg2g001440*, *Cg9g003720*, and *Cg5g039010*) were upregulated. A total of 7 DEGs related to glutathione S-transferase were all found to be upregulated, including *Cg5g036980*, *Cg1g024200*, *Cg7g012400*, *Cg6g008130*, *Cg6g003160*, *Cg5g002950*, and *Cg2g020810*. Additionally, 3 DEGs associated with APX were identified, of which 2 genes (*Cg4g002260* and *Cg1g001450*) were downregulated and 1 gene (*Cg6g002810*) was upregulated. Furthermore, 1 DEG related to ferritin CAT was downregulated (*Cg9g000870*), and 1 DEG associated with glutathione peroxidase was upregulated (*Cg5g002990*). Lastly, 1 DEG associated with superoxide dismutase was downregulated (*Cg8g018870*).

### 2.8. Identification of Metal Transporter-Related Genes

A total of 39 DEGs related to metal transport were identified in the leaves of Shatian pomelo (Table S8). This includes 19 DEGs associated with zinc finger proteins, with 8 genes upregulated and 11 downregulated. Four DEGs associated with chloroplast magnesium chelatase were identified, of which two were upregulated (*Cg9g021280* and *Cg2g004190*) and two downregulated (*Cg2g043060* and *Cg2g004200*). Additionally, four DEGs related to calcium transport ATPase were identified, with three downregulated (*Cg3g008610*, *Cg1g006790*, and *Cg5g012700*) and one upregulated (*Cg3g010620*). All three identified phosphate transporters were downregulated (*Cg6g017660*, *Cg6g025180*, and *Cg7g015130*). Among the three DEGs associated with metal tolerance proteins, two were downregulated (*Cg9g029250* and *Cg1g012170*) and one was upregulated (*Cg7g005360*). All three *calc-binding proteins* showed downregulation (*Cg2g022120*, *Cg2g041040*, and *Cg2g022150*). One *Al-sensitive protein* (*Cg6g003330*) was upregulated, whereas one *inorganic phosphate transporter* (*Cg5g034040*) and one *sulfate transporter* (*Cg5g035050*) were downregulated.

### 2.9. Identification of Transcription Factor-Related Genes

In the leaves of Shatian pomelo, 66 DEGs related to transcription factors were identified (Table S9). This includes 9 DEGs associated with bHLH1 transcription factors, with 2 genes upregulated (*Cg5g026370* and *Cg1g007400*) and 7 downregulated. Twenty-six DEGs were associated with MYB transcription factors, comprising 11 upregulated and 15 downregulated. Additionally, there were 20 *ethylene response transcription factors*, of which 14 were upregulated and 6 downregulated. Five *GATA transcription factors* were downregulated, specifically *Cg5g031000*, *Cg1g004300*, *Cg5g043880*, *Cg1g014090*, and *Cg4g006690*. Four *transcription inhibitors* were also downregulated (*Cg5g035280*, *Cg1g023660*, *Cg8g013110*, and *Cg6g014790*). Moreover, two *nuclear transcription factors* were upregulated (*Cg7g023360* and *Cg9g021740*).

### 2.10. qRT-PCR Verification of Gene Expression

**Figure 8:**
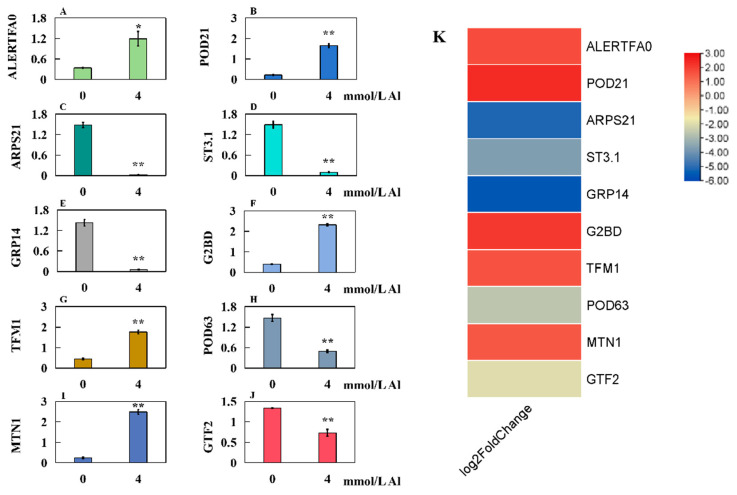
Relative expression levels of 10 genes in leaves of Shatian pomelo under Al toxicity stress for 20 days: (**A**) *ALERTFA0* (*AP2-like ethylene-responsive transcription factor At2g41710*), (**B**) *POD21* (*Peroxidase 21*), (**C**) *ARPS21* (*Auxin-responsive protein SAUR21*), (**D**) *ST3.1* (*Sulfate transporter 3.1*), (**E**) *GRP14* (*Gibberellin-regulated protein 14*), (**F**) *G2BD* (*Gibberellin 2-beta-dioxygenase*), (**G**) *TFM1* (*Transcription factor MYB1*), (**H**) *POD63* (*Peroxidase 63*), (**I**) *MTN1* (*Metal transporter Nramp1*), and (**J**) *GTF2* (*GATA transcription factor 2*). (**K**) The heatmap of log2FC values for the transcriptome of Shatian pomelo leaves. The results were expressed as the mean and standard deviation of three repeated biological experiments. Student’s *t*-test was used to test the significance of different concentrations between the control group and the Al poisoning group. An asterisk on the bar chart indicated a significant difference (* *p* < 0.05) or a very significant difference (** *p* < 0.01) between the data.

To verify gene expression in Shatian pomelo leaves under Al toxicity stress, the expression levels of 10 genes associated with hormones, antioxidant enzymes, ion transporters, and transcription factors were analyzed. The results indicated that compared to the control group (0 mmol/L Al), the relative expressions of *ALERTFA0*, *POD21*, *G2BD*, *TFM1*, and *MTN1* in the experimental group (4.0 mmol/L Al) significantly increased (Figure 8A,B,F,G,I). Conversely, the relative expression levels of *ARPS21*, *ST3.1*, *GRP14*, *POD63*, and *GTF2* significantly decreased (Figure 8C–E,H,J). Notably, the qRT-PCR results aligned with those obtained from transcriptome sequencing.

## 3. Discussion

Aluminum toxicity, prevalent in acidic soils, significantly restricts plant growth by inhibiting cell expansion and division in leaves [24]. This inhibition directly affects leaf development [12]. Additionally, gamma-aminobutyric acid (GABA) plays a crucial role in regulating plant responses to abiotic stresses, including Al stress, by modulating the transport of malic acid through membrane channels [25]. In barley (*Hordeum vulgare*), the cell wall is associated with 85%–90% of the total accumulated Al, which influences the composition of the cell wall by causing changes in the cell wall polysaccharides. Al stress leads to the accumulation of hemicellulose and polysaccharides, which hardens and thickens the cell wall [26]. Due to the decrease in fresh weight and the relative stability or decrease in dry weight, the FW/DW ratio will decrease, reflecting the decrease in water content in plants, which is a physiological response of plants to stress [27]. Observations showed that Al poisoning inhibited the development of Shatian pomelo leaves, leading to reductions in both dry and fresh weight, as well as leaf surface area and perimeter. This indicates that high concentrations of Al stress markedly inhibited the growth of Shatian pomelo leaves. The FW/DW ratio of leaves showed a decreasing trend under 0 to 8 mM Al stress (Table S10), indicating that the water content of Shatian pomelo leaves decreased under Al stress. Similar findings have been reported in other plant species; for instance, Al stress has been shown to hinder leaf growth in peanuts, resulting in the differential expression of numerous genes [13]. Therefore, the inhibition of leaf growth in Shatian pomelo under Al toxicity stress may involve complex interactions regulated by multiple genes.

Previous studies have demonstrated that an excess of Al in soil can result in irreversible damage to plants [28]. Al stress can lead to increased levels of reactive oxygen species (ROS) in plants, resulting in oxidative stress, damage to cell membrane structure and chlorophyll molecules, and lead to leaf greening and function impairment [15]. Under Al stress, plants generate ROS, leading to oxidative stress, whereas antioxidant enzymes such as SOD, POD, and CAT play crucial roles in detoxifying excess ROS and shielding plant cells from oxidative damage [27]. Furthermore, PRO and MDA serve as biomarkers for oxidative stress, with alterations in their levels reflecting the extent of damage to the plant cell membrane system [27]. For instance, the overexpression of GASR1 in rice (*Oryza sativa*) enhances Al tolerance without influencing the distribution and accumulation of Al, while simultaneously promoting the production of ROS in the plant [29]. Modulating the expression and activity of antioxidant enzymes can effectively remove H_2_O_2_ under Al stress, thereby improving soybean resistance to Al [30]. In our study, the observed increase in the activity of APX, CAT, POD, and SOD likely aids the leaves of Shatian pomelo in mitigating the damage caused by ROS generated under Al stress, thereby alleviating the inhibitory effects of Al on leaf growth. Additionally, as Al concentration increased, MDA content in Shatian pomelo leaves also increased. The elevation of MDA levels under Al stress indicates that plant cell membranes are experiencing oxidative stress, resulting in lipid peroxidation, which adversely affects membrane structure and function [31]. The antioxidant defense system in the leaves of Shatian pomelo could effectively reduce the accumulation of ROS and free radicals, thus minimizing the damage inflicted by Al stress on these plants.

Osmoregulatory substances, such as soluble sugars in plant cells, play a vital role in maintaining osmotic balance [32]. These substances enhance the water retention capacity of cells, uphold cellular structural stability, and alleviate physiological and metabolic imbalances induced by stress [32]. In our study, the notable increase in soluble sugars suggests that the accumulation of soluble proteins in Shatian pomelo leaves contributes to the mitigation of physiological and metabolic disruptions resulting from Al stress.

This study revealed a significant accumulation of Al in Shatian pomelo leaves, concurrent with decreased levels of B, Ca, Mg, Mn, and Fe. In acidic soils, Al competes with Mg for binding sites on transport channels, thereby inhibiting Mg uptake and increasing plant sensitivity to Al toxicity [33]. For instance, disrupting the magnesium transporter gene *OsMGT1* in rice resulted in reduced Mg uptake and increased Al sensitivity [34]. Furthermore, Al stress can activate tolerance gene expression in plants, which is pivotal in the transport of various ions. The absorption of Al by plants may compete with that of Ca, Fe, and Mn, among others [18]. Consequently, the observed decrease in B, Ca, Mg, Mn, and Fe in Shatian pomelo leaves during this experiment may be attributed to competitive absorption dynamics in the context of Al-induced stress.

Auxin, a fundamental plant hormone, plays a critical role at every stage of leaf development and contributes significantly to leaf pattern formation and morphological shaping [35]. Auxin plays a key role in leaf morphogenesis [36]. It affects the size and shape of leaves by regulating cell division and extension [36]. Studies have shown that the aggregation of auxin transport protein PIN1 on both sides of the leaf primordium can promote the accumulation of auxin at the leaf margin, thus activating the *SlLAM1* expressed at the leaf margin and making the leaf primordium develop from bilateral symmetry into flat leaves [36]. Quantitative PCR results also showed that genes related to auxin synthesis were involved in plant response to Al toxicity. Under Al toxicity stress, genes related to auxin in Shatian pomelo leaves showed a downward trend. SAUR gene family is a member of the auxin early response gene family and is one of the indispensable regulatory factors in the auxin signal transduction pathway [37]. In *Agave americana* L., the expression of *SAUR* is significantly increased in undeveloped leaves and significantly decreased in developed leaves, indicating that *SAUR* plays a key role in leaf development [38]. In the qRT-PCR verification of this study, the expression of auxin reactive protein gene *SAUR21* was significantly reduced, indicating that the growth and development of leaves were hindered by Al stress, which hindered auxin synthesis. In addition, *AP2/ERF transcription factors* enhance plant tolerance to abiotic stress by regulating downstream gene expression, independent of ABA signal transduction pathways [39]. For example, overexpression of tomato *ERF transcription factor SlERF84* in transgenic plants can enhance plant tolerance to drought and salt stress [39]. The expression of *ALERTFA0* was significantly increased in the leaves, and the resistance of Shatian pomelo to Al stress might be enhanced by directly regulating downstream genes.

Notably, the expression of *POD21* in Shatian pomelo leaves increased substantially following Al stress. This upregulation of peroxidase activity may indicate that Shatian pomelo activates its defense mechanisms in response to Al stress. By enhancing the activity of antioxidant protective enzymes, the plant can mitigate the damage caused by ROS, thereby maintaining membrane stability and ensuring the physiological and metabolic balance of the plant [13]. Gibberellin (GAs) plays an important role in cell elongation and leaf expansion during plant growth and development [40]. One study found that the reduction in cell length of the leaf defect phenotype was mainly caused by GA deficiency, resulting in plant dwarfing [41]. Gibberellin 2-β-dioxygenase (GA2oxs) is a crucial enzyme in the gibberellin metabolic pathway, which regulates gibberellin levels in plants by decomposing active gibberellins [42]. GA2oxs plays an important role in plant response to abiotic stress [43]. Overexpression of *GA2oxs* in tomato (*Solanum lycopersicum* L.) will lead to increased resistance to abiotic stress, decreased expression of the gibberellin synthase gene, and significantly decreased content of active gibberellin [44]. By increasing the expression of *GA2oxs*, the level of gibberellin in Shatian Pomelo was decreased, and the tolerance to Al toxicity was improved, while the lower gibberellin was not conducive to leaf morphogenesis. *MYB transcription factor MsMYB741* regulates flavonoid biosynthesis in *Medicago sativa* L., thereby improving resistance to Al toxicity [45]. Specifically, overexpression of *MsMYB741* can significantly reduce the Al content in plants and improve the Al toxicity resistance of transgenic *Medicago sativa* lines [45]. By positively regulating the expression of *MsPAL* and *MsCHI*, *MsMYB741* promotes the accumulation of total flavonoids and increases the secretion of flavonoids in *Medicago sativa* roots [45]. On the one hand, the active oxygen species caused by Al stress can be removed, and the oxidative damage of the plasma membrane system can be reduced; on the other hand, excessive Al content in plants can be reduced by increasing the secretion of root flavonoids to chelate Al [45]. In this study, the expression level of transcription factor *MYB1* in the leaves of Shatian pomelo was significantly increased, indicating that plants improved their tolerance to Al stress by enhancing the expression of *MYB transcription factor*.

The upregulation of *Nramp1* may assist plants in maintaining metal ion homeostasis within their cells. In barley (*Hordeum vulgare*), NRAMP family genes are positively regulated under heavy metal ion stress, and the expression level of *HvNramp1* is significantly increased under manganese stress, thus reducing the toxic effect of manganese ion on plants [46]. In addition, metal transporter NRAMP transports Al into cells and isolates it in vacuoles by reducing the accumulation of Al in cell walls, thus participating in the regulation of Al resistance in rice (*Oryza sativa*) [47]. In this experiment, the expression of *MTN1* (*metal transporter Nramp1*) was significantly increased under Al toxicity, which was conducive to reducing the toxic effect of Al stress on Shatian pomelo leaves.

**Figure 9:**
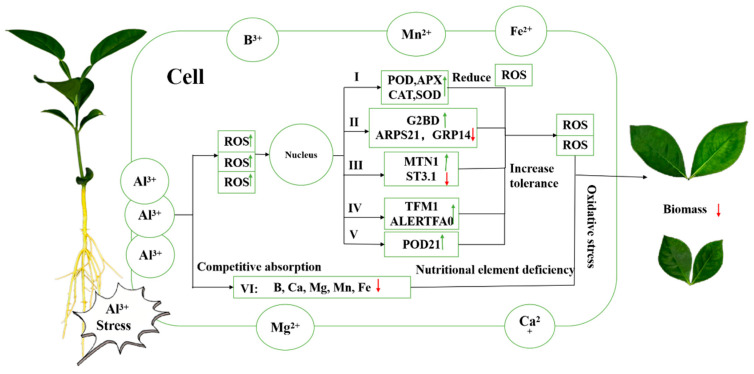
Regulatory pathway diagram of Shatin pomelo leaves responding to Al-poisoning stress. I: Antioxidant enzymes; II: Genes related to hormone synthesis; III: Ion transporter-related genes; IV: Transcription factor-related genes; V; Peroxidase-related gene; VI: Ion. Small green arrows indicated upregulated gene expression, increased substance content, or enzyme activity. Small red arrows indicated downregulated gene expression or reduced substance content.

Under Al toxicity stress, the content of Al in Shatian pomelo leaves increased sharply, and the high concentration of Al competed with B, Ca, Mg, Mn, Fe, and other elements for binding sites, resulting in a decrease in the content of these five elements in Shatian pomelo leaves. At the same time, due to the toxic effect of Al stress, the content of reactive oxygen species (ROS) in plants increased, which caused oxidative stress in leaf cells, which led to increased membrane damage and increased the content of malondialdehyde (MDA). A series of changes in the leaf cells of Shatian pomelo activated upregulated or downregulated the expression of genes related to hormones, antioxidant enzymes, transporters, and transcription factors, such as *ALERTFA0*, *POD21*, *ARPS21*, *ST3.1*, *GRP14*, *G2BD*, *TFM1*, and *MTN1*. Finally, due to oxidation stress and nutrient deficiency caused by Al stress, leaf growth was severely inhibited and biomass decreased significantly (Figure 9). These results indicated that Al stress inhibited the growth of Shatian pomelo leaves by inducing the regulation of plant hormones, transcription factors, ion transporters, and other mechanisms. The specific regulatory pathways need to be further studied.

## 4. Materials and Methods

### 4.1. Plant Materials and Hydroponic Treatment

The plant material utilized in this study is Guangxi Shatian Pomelo, cultivated by Sanhao Ecological Agriculture in Public Security Town, Zhongshan County, Hezhou City, in the Guangxi Zhuang Autonomous Region of China. The plants were cultured in the plant growth room at the Coastal Agricultural Sciences College, Guangdong Ocean University (E: 110.30311, N: 21.15005). Shatian pomelo seeds were selected based on intact seed coats and uniform size; the outer seed coats were removed, and the seeds were transferred to moist quartz sand for germination over a period of 20 days. The uniformly developed Shatian pomelo plants were subsequently transferred to a 5 L plastic bucket, where they were provided with a modified Hoagland nutrient solution for hydroponic culture [48]. The composition of the hydroponic nutrient solution is detailed in Table S11. The electrical conductivity (EC) and pH of the nutrient solution were about 1340 µs/cm and 4, respectively. All chemical reagents employed in this study were of analytical grade (Kermel, Tianjin, China).

Al_2_(SO_4_)_3_·18H_2_O (Shanghai Reagent, Shanghai, China) was utilized as a source of Al ions and was incorporated into the hydroponic nutrient solution for Shatian pomelo at concentrations of 0, 1.0, 2.0, 4.0, and 8.0 mmol/L for treatment. The control group received no Al (0 mmol/L), and each treatment was replicated three times. Shatian pomelo plants were maintained under the following conditions: daytime temperatures ranged from 23–28 °C, night temperatures were between 20 and 22 °C, light intensity was set at 2000 lux, and the light period was approximately 12 h per day. The nutrient solution was refreshed every 5 days. After a 20-day treatment period, leaves (counting the first pair of fully unfolded leaves from top to bottom) from the Shatian pomelo plants were harvested.

### 4.2. Determination of Dry and Fresh Weight of Shatian Pomelo Leaves

The fresh weight of each Shatian pomelo leaf was measured using a BS124S electronic scale (Sartorius, Göttingen, Germany). Subsequently, the leaf samples were transferred to a constant temperature oven with electric blast air (Yiheng, Shanghai, China) and dried at 75 °C for 7 days. The dry weight of each treated sample was recorded, with three biological replicates performed [49].

### 4.3. Determination of Morphological Indexes of Shatin Pomelo Leaves

Morphological characteristics of the Shatian pomelo leaves were documented using leaf morphology and lesion analysis (Topu Yunnong, Hangzhou, Zhejiang, China), with photographs taken post-Al treatment. The total area and perimeter of the blade were analyzed. These measurements were repeated three times.

### 4.4. Determination of Physiological Response Indexes of Shatin Pomelo Leaves

Shatian pomelo plants were subjected to varying concentrations of Al (0, 1.0, 2.0, 4.0, and 8.0 mmol/L) for a duration of 20 days, following which leaves were collected. Six physiological responses were quantified using methods outlined in Shi et al. 2024 [13]. SOD activity was assessed via the nitrogen blue tetrazole method. POD activity was measured using the guaiacol method. CAT activity was determined with a spectrophotometer (Yuexi UV5100B, Shanghai, China). Soluble protein content was quantified using the Coomassie Brilliant Blue staining technique, MDA content was established through the thiobarbituric acid method, and APX activity was measured according to the experimental techniques described by Shi et al. 2024 [13].

### 4.5. Determination of Ion Content in Shatin Pomelo Leaves

Leaves of Shatian pomelo were treated with 0 mmol/L (control group) and 4.0 mmol/L AL (Al toxic treatment group), respectively. Leaf samples (0.15 g) were dried and subsequently dissolved in nitric acid (68%) (Guangzhou Reagent, Guangzhou, China). The contents of B, Mg, Al, Ca, Mn, and Fe in the leaves of Shatian pomelo were analyzed using an Agilent 7500cx ICP-MS (Agilent, Santa Clara, CA, USA), with three biological replicates conducted [50].

### 4.6. Library Construction and Transcriptome Sequencing Analysis

Fresh leaf samples of Shatian pomelo seedlings were treated with either 0 mmol/L control (group) or 4.0 mmol/L Al for a duration of 20 days. Total RNA (*ribonucleic acid*) was extracted from the leaf samples to construct an mRNA library for Shatian pomelo seedlings. Biological repeats were conducted three times for each sample. RNA extraction was performed using the ultra-pure RNA kit (CWBIO, Nanjing, China). One microgram of the extracted total RNA was utilized for poly (A) mRNA separation, mRNA fragmentation, and cDNA synthesis, followed by PCR amplification and verification. The mRNA library construction and sequencing were carried out on the Illumina HiSeq platform from Suzhou Panomic Biomedical Technology Co., Ltd (Suzhou, Jiangsu, China). Fastq format files were processed using Cutadapt (V1.9.1) to obtain clean data. The reference genome sequence was downloaded from UCSC, NCBI, ENSEMBL, and other genome databases. Hisat2 (v2.2.1) was employed to align the clean data to the reference genome. Gene expression levels were estimated using HTSeq (v0.6.1) [51], whereas the DESeq2 package was used to identify DEGs [52]. Go (gene ontology) terms for significantly enriched genes were determined using the GOseq tool (v1.34.1) [53]. KEGG pathway enrichment analysis was conducted with version 3.4.4 software. The resultant data were subsequently submitted to the NCBI database under the accession number PRJNA1182287.

### 4.7. qRT-PCR Detection

The hyperpure RNA extraction kit (CWBIO, Shanghai, China) was utilized to extract ribonucleic acid (RNA) from Shatian pomelo leaves. After eliminating genomic DNA, cDNA was synthesized using a reverse transcription kit (Takara, Maebashi, Japan). Real-time fluorescence quantitative PCR (Bio-rad Company, Hercules, CA, USA) was employed for qRT-PCR analysis [54]. The gene *Cg2g039090* (*β-tubulin*) served as a reference gene, and the relative transcription levels of the target gene were calculated against this internal reference gene [12]. Primers used for qRT-PCR analysis are shown in Table S12.

### 4.8. Data Analysis

Statistical analysis was performed using Microsoft Excel 2010 (Microsoft, Redmond, WA, USA) and SPSS (Statistical Package for Social Science) version 19.0 software (IBM Corporation, New York, NY, USA). Student’s *t*-test was utilized for statistical comparison and significance analysis between the two groups of data, whereas the Waller–Duncan test was employed to assess the significance of differences among multiple groups of data [55].

## 5. Conclusions

The physiological and molecular reaction mechanism of Shatian pomelo under Al stress was studied. Excessive accumulation of Al can increase the active oxygen activity in Shatian pomelo, damage the cell membrane structure, and lead to leaf damage. The increase in Al content in plants will destroy the balance of metal ion absorption and transport and inhibit the synthesis of plant hormones, such as auxin and gibberellin, which will lead to the growth and development of leaves. Transcriptome sequencing revealed 4868 DEGs in the leaves under Al poisoning conditions, with 1994 DEGs being upregulated and 2874 DEGs downregulated. Furthermore, 10 of these DEGs, which are associated with ion transporters, hormones, antioxidant enzymes, and transcription factors, were validated through qRT-PCR, with results consistent with the transcriptome sequencing data. These findings contribute to a deeper understanding of how Shatian pomelo leaves respond to Al poisoning and provide a foundational basis for further exploration of specific molecular regulatory mechanisms.

## Data Availability

Data are contained within the article and Supplementary Materials.

## Supplementary Materials

The following supporting information can be downloaded at https://www.mdpi.com/article/10.3390/plants14040603/s1, Table S1: *Citrus maxima “Shatian Yu”* leaf sample sequencing raw data quality statistics; Table S2: *Citrus maxima “Shatian Yu”* leaf sample sequencing clean data quality statistics; Table S3: Statistics of expressed genes identified in the leaf of *Citrus maxima “Shatian Yu”*; Table S4: GO enrichment analysis of *Citrus maxima “Shatian Yu”* leaves; Table S5: KEGG analysis of DEGs in *Citrus maxima “Shatian Yu”* leaves; Table S6: Identification of DEGs related to hormone in leaves of *Citrus maxima “Shatian Yu”* leaves under Al stress; Table S7: Identification of DEGs associated with antioxidant enzymes in leaves of *Citrus maxima “Shatian Yu”* leaves under Al stress; Table S8: Identification of DEGs related to metal transporter in leaves of *Citrus maxima “Shatian Yu”* leaves under Al stress; Table S9: Identification of DEGs related to transcription factor in leaves of *Citrus maxima “Shatian Yu”* leaves under Al stress; Table S10: Shatian pomelo leaf ratio of fresh weight/dry weight; Table S11: Composition and content in hydroponic solution; Table S12: Primers used for qPCR in the study.
