# Supplementary material for: Reaction Mechanism of Aluminum Toxicity on Leaf Growth of Shatian Pomelo Seedlings
Source: Plants (Basel). 2025 Feb 17;14(4):603. doi: 10.3390/plants14040603 (PMC11859590; doi:10.3390/plants14040603)
Supplement: Supplementary file 1 [file plants-14-00603-s001.zip › Tables S2-S12-2024.12.31.pdf]

Table S2. ***Citrus maxima 'Shatian Yu'*** **leaf sample sequencing clean data quality statistics**

Sample	Length	Reads	Bases	Q20 (%)	Q30 (%)	GC (%)	N (ppm)
L0-1	146.64	36139422	5299631969	98.32	94.92	43.98	4.72
L0-2	146.31	44647708	6532607457	98.35	94.98	44.00	5.17
L0-3	146.45	50308642	7367931690	98.15	94.54	44.27	4.79
L4-1	146.08	38935598	5687581871	98.31	94.87	43.67	1.54
L4-2	146.38	42026500	6151637422	98.26	94.78	43.81	4.70

Table S3. Statistics of expressed genes identified in the leaf of ***Citrus maxima 'Shatian Yu'***

Control-VS-Treatment	All Gene Expression	Total DEGs	Up DEGs	Down DEGs
L4-VS-L0	30123	4868	1994	2874

Table S4. GO enrichment analysis of *Citrus maxima 'Shatian Yu'* leaves

Category	GO:ID	Term	Up	Down	DEG	Total	pvalue	FDR
CC	GO:0005576	extracellular region	37	171	208	690	3.79047E-16	2.48541E-12
BP	GO:0009834	secondary cell wall biogenesis	0	35	35	53	1.74286E-13	5.71396E-10
MF	GO:0008017	microtubule binding	0	51	51	108	7.72771E-11	1.68902E-07
CC	GO:0005886	plasma membrane	183	323	506	2091	1.1266E-10	1.84678E-07
BP	GO:0007018	microtubule-based movement	0	31	31	55	1.16832E-09	1.53214E-06
MF	GO:0003777	microtubule motor activity	0	29	29	52	4.94632E-09	2.48541E-12
BP	GO:0009768	photosynthesis, light harvesting in photosystem I	0	15	15	19	2.77298E-08	5.71396E-10
MF	GO:0022857	transmembrane transporter activity	36	29	65	171	2.79033E-08	1.68902E-07
CC	GO:0048046	apoplast	17	83	100	360	6.7734E-08	1.84678E-07
BP	GO:0071555	cell wall organization	8	69	77	228	1.2387E-07	1.53214E-06
BP	GO:0044772	mitotic cell cycle phase transition	0	15	15	20	1.5572E-07	5.40551E-06
CC	GO:0009579	thylakoid	4	33	37	92	2.08311E-06	2.28702E-05
CC	GO:0005874	microtubule	0	48	48	131	2.59895E-06	2.28702E-05
CC	GO:0009534	chloroplast thylakoid	7	41	48	134	5.41778E-06	4.9348E-05
BP	GO:0009664	plant-type cell wall organization	2	17	19	37	5.86624E-06	2.48541E-12
BP	GO:0009416	response to light stimulus	10	36	46	132	1.02912E-05	5.71396E-10
MF	GO:0004553	hydrolase activity, hydrolyzing O-glycosyl compounds	4	13	17	32	1.19723E-05	1.68902E-07
BP	GO:0055085	transmembrane transport	26	29	55	162	1.34832E-05	1.84678E-07
BP	GO:0009734	auxin-activated signaling pathway	17	46	63	215	1.66574E-05	1.53214E-06
CC	GO:0009505	plant-type cell wall	16	61	77	263	1.90851E-05	5.40551E-06
BP	GO:0010411	xyloglucan metabolic process	2	15	17	33	1.95616E-05	2.28702E-05
MF	GO:0016538	cyclin-dependent protein serine/threonine kinase regulator activity	0	15	15	26	2.33465E-05	2.28702E-05
MF	GO:0015293	symporter activity	15	15	30	71	2.51375E-05	4.9348E-05
MF	GO:0016762	xyloglucan:xyloglucosyl transferase activity	2	12	14	26	3.67526E-05	8.12217E-05
BP	GO:0010597	green leaf volatile biosynthetic process	6	10	16	30	3.83787E-05	9.28233E-05
BP	GO:0016042	lipid catabolic process	8	29	37	106	5.20524E-05	0.001138244
MF	GO:0042910	xenobiotic transporter activity	18	5	23	51	5.29769E-05	0.001310869
BP	GO:0000079	regulation of cyclin-dependent protein serine/threonine kinase activity	1	14	15	28	6.13436E-05	0.002537458
MF	GO:0003700	sequence-specific DNA binding transcription factor activity	93	118	211	888	6.30739E-05	0.002564329
BP	GO:1990961	drug transmembrane export	15	5	20	43	8.58707E-05	0.004217459

Table S5. KEGG analysis of DEGs in *Citrus maxima 'Shatian Yu'* leaves

Pathway ID	Pathway	Up	Down	DEGs	Total	Pvalue	Qvalue
ko00511	Other glycan degradation	3	7	10	30	0.008425714	0.03763486
ko01100	Metabolic pathways	265	326	591	2779	2.84909E-15	3.81777E-13
ko00196	Photosynthesis - antenna proteins	0	15	15	19	3.06486E-10	1.36897E-08
ko00053	Ascorbate and aldarate metabolism	6	11	17	49	0.000814513	0.006063594
ko00945	Stilbenoid, diarylheptanoid and gingerol biosynthesis	9	5	14	38	0.000927817	0.006543554
ko00590	Arachidonic acid metabolism	10	2	12	29	0.000478805	0.004218866
ko04075	Plant hormone signal transduction	25	41	66	249	5.94744E-05	0.000664131
ko00906	Carotenoid biosynthesis	4	9	13	43	0.01029161	0.04448632
ko00520	Amino sugar and nucleotide sugar metabolism	19	27	46	147	6.99082E-06	0.000105294
ko00910	Nitrogen metabolism	5	5	10	29	0.006294108	0.03123742
ko00940	Phenylpropanoid biosynthesis	28	25	53	176	5.88844E-06	0.000105294
ko03030	DNA replication	6	11	17	58	0.006799824	0.03254201
ko01212	Fatty acid metabolism	12	8	20	74	0.01110784	0.04651409
ko00073	Cutin, suberine and wax biosynthesis	2	10	12	28	0.000312897	0.002994867
ko00904	Diterpenoid biosynthesis	13	4	17	30	1.63377E-07	4.37849E-06
ko00030	Pentose phosphate pathway	16	5	21	60	0.000228958	0.00236003
ko00591	Linoleic acid metabolism	3	8	11	19	9.71809E-06	0.000130222
ko00010	Glycolysis / Gluconeogenesis	24	7	31	119	0.004981756	0.0256752
ko01110	Biosynthesis of secondary metabolites	180	161	341	1442	1.57924E-13	1.05809E-11
ko00966	Glucosinolate biosynthesis	3	4	7	17	0.004195436	0.02248754
ko00531	Glycosaminoglycan degradation	3	7	10	26	0.002311007	0.01414944
ko04814	Motor proteins	0	41	41	101	5.35904E-09	1.79528E-07
ko00860	Porphyrin metabolism	8	15	23	50	4.48041E-07	1.00063E-05
ko00061	Fatty acid biosynthesis	9	6	15	50	0.007571556	0.03498581
ko00380	Tryptophan metabolism	13	3	16	49	0.002323043	0.01414944
ko00051	Fructose and mannose metabolism	8	14	22	67	0.000503745	0.004218866
ko04626	Plant-pathogen interaction	52	36	88	382	0.001006061	0.00674061
ko00500	Starch and sucrose metabolism	17	18	35	136	0.003988617	0.02248754
ko01200	Carbon metabolism	49	17	66	269	0.000674024	0.005312897

Table S6. Identification of DEGs related to hormone in leaves of *Citrus maxima 'Shatian Yu'* leaves under Aluminum stress

Gene ID	log2FoldChange	Description
Cg1g010480	-3.47	Auxin efflux carrier component 5
Cg6g006550	-1.75	Auxin efflux carrier component 6
Cg9g006770	-1.71	Auxin efflux carrier component 8
Cg4g020590	-1.37	Auxin response factor 19
CgUng005460	-4.55	Auxin response factor 2B
CgUng005390	-4.75	Auxin response factor 3
Cg2g009950	-1.02	Auxin response factor 9
Cg3g010270	-5.19	Auxin transporter-like protein 1
Cg7g001220	-1.18	Auxin transporter-like protein 2
Cg1g015920	-1.42	Auxin transporter-like protein 3
Cg1g015910	-2.32	Auxin transporter-like protein 5
CgUng005220	-5.2	Auxin-binding protein ABP19a
Cg3g016080	-2.12	Auxin-induced protein 15A
Cg8g001890	-3.58	Auxin-induced protein 15A
CgUng005470	1.86	Auxin-induced protein 22D
Cg5g040580	1.32	Auxin-induced protein 6B
CgUng005210	-5.79	Auxin-induced protein AUX22
Cg1g012560	-2.26	Auxin-induced protein X10A
Cg8g001020	-8.85	Auxin-induced protein X10A
Cg6g001170	1.33	Auxin-responsive protein IAA1
Cg8g000980	-8.6	Auxin-responsive protein IAA1
Cg5g036600	7.21	Auxin-responsive protein IAA14
Cg2g035770	-4.63	Auxin-responsive protein IAA27
Cg3g023820	-1.5	Auxin-responsive protein IAA29
CgUng005420	-7.5	Auxin-responsive protein IAA30
Cg3g017880	-1.84	Auxin-responsive protein IAA32
Cg8g001010	-6.33	Auxin-responsive protein SAUR20
Cg4g002180	2.07	Auxin-responsive protein SAUR21
Cg3g001600	1.49	Auxin-responsive protein SAUR24
Cg8g000990	-6.66	Auxin-responsive protein SAUR50
CgUng005430	-6.44	Auxin-responsive protein SAUR63
Cg8g001030	-6.47	Auxin-responsive protein SAUR64
Cg6g017830	-1.68	Auxin-responsive protein SAUR64
CgUng005200	-6.12	Auxin-responsive protein SAUR64
Cg4g018030	-2.08	Auxin-responsive protein SAUR64
Cg5g039360	3.05	Auxin-responsive protein SAUR72
Cg9g028950	-1.3	Auxin-responsive protein SAUR76
Cg4g008150	-2.15	Probable auxin efflux carrier component 1c
Cg6g023900	-1.55	Protein AUXIN SIGNALING F-BOX 2

CgUng005410	-4.19	Protein SMALL AUXIN UP-REGULATED RNA 51
Cg5g010670	-1.65	Protein IMPAIRED IN BABA-INDUCED STERILITY 1
Cg8g018300	2.36	GABA transporter 1
Cg5g001590	-1.14	Probable GABA transporter 2
Cg6g023440	-5.57	Gibberellin-regulated protein 14
Cg6g024880	-4.62	Gibberellin-regulated protein 4
Cg3g017400	-3.95	Gibberellin 20 oxidase 2
Cg5g010180	-3.6	Gibberellin-regulated protein 6
Cg9g026010	-2.87	Gibberellin 20 oxidase 1
Cg2g040470	-2.83	Gibberellin 2-beta-dioxygenase 8
Cg6g018040	-1.84	Gibberellin-regulated protein 5
Cg3g020210	-1.5	Gibberellin-regulated protein 9
Cg5g016200	1.03	Gibberellin 2-beta-dioxygenase 1
Cg2g030540	1.11	Gibberellin receptor GID1C
Cg5g042980	1.45	Gibberellin 20 oxidase 2
Cg2g022960	1.75	Chitin-inducible gibberellin-responsive protein 1
Cg2g044860	2.12	Gibberellin 2-beta-dioxygenase
Cg2g023660	2.66	Gibberellin 2-beta-dioxygenase 8
Cg5g036390	3.13	Gibberellin receptor GID1B
Cg7g013330	4.1	Gibberellin 2-beta-dioxygenase 6
Cg4g008100	6.59	Gibberellin 3-beta-dioxygenase 3

Table S7. Identification of DEGs associated with antioxidant enzymes in leaves of *Citrus maxima 'Shatian Yu'* leaves under Aluminum stress

Gene ID	log2FoldChange	Description
Cg2g001370	1.24	Peroxidase 15
Cg2g001400	-1.15	Peroxidase 15
Cg2g001440	3.62	Peroxidase 15
Cg6g023390	-2.6	Peroxidase 63
Cg2g018020	-1.79	Peroxidase 3
Cg2g006540	-2.54	Peroxidase 4
Cg7g014170	-4.59	Peroxidase 65
Cg1g004840	-3.25	Peroxidase 72
Cg1g001240	-2.6	Peroxidase 64
Cg3g024510	-5.25	Peroxidase 64
Cg3g018770	-1.3	Peroxidase 12
Cg1g009400	2.4	Peroxidase 21
Cg1g008440	-1.59	Peroxidase 17
Cg9g003720	4.4	Peroxidase 24
Cg2g042930	-5.15	Peroxidase 19
Cg5g039010	5.2	Peroxidase 5
Cg5g004520	-3.93	Peroxidase 65
Cg8g005280	1.33	Peroxidase 10
Cg5g036980	1.7	Glutathione S-transferase F8, chloroplastic
Cg1g024200	1.02	Glutathione S-transferase U7
Cg7g012400	3.3	Glutathione S-transferase F9
Cg6g008130	1.32	Glutathione S-transferase U7
Cg6g003160	1.41	Glutathione S-transferase L3
Cg5g002950	1.71	Glutathione S-transferase T1
Cg2g020810	1.68	Glutathione S-transferase L3
Cg4g002260	-1.08	Putative L-ascorbate peroxidase 6
Cg6g002810	1.59	L-ascorbate peroxidase 2, cytosolic
Cg1g001450	-1.16	L-ascorbate peroxidase 3
Cg9g000870	-2.48	Ferritin-like catalase Nec2
Cg5g002990	2.19	Probable phospholipid hydroperoxide glutathione peroxidase
Cg8g018870	-1.86	Superoxide dismutase [Cu-Zn], chloroplastic

Table S8. Identification of DEGs related to metal transporter in leaves of *Citrus maxima 'Shatian Yu'* leaves under Aluminum stress

Gene ID	log2FoldChange	Description
Cg1g006080	-6.93	Zinc finger protein 1
Cg1g021060	-2.09	Zinc finger protein 2
Cg5g003670	-1.26	Zinc finger protein BRUTUS-like At1g18910
Cg2g010020	1.67	Zinc finger protein CONSTANS-LIKE 10
Cg5g022770	-1.72	Zinc finger protein CONSTANS-LIKE 6
Cg1g003910	1.23	Zinc finger protein ENHYDROUS
Cg2g008170	1.05	Zinc finger protein MAGPIE
Cg5g036880	1.31	Zinc finger protein MAGPIE
Cg2g028650	-1.31	Zinc finger protein SHOOT GRAVITROPISM 5
Cg7g023230	1.1	Zinc finger protein WIP2
Cg5g043800	-1.13	Zinc finger protein ZAT9
Cg2g035580	-3.59	Zinc finger CCCH domain-containing protein 14
Cg6g019160	-2.35	Zinc finger CCCH domain-containing protein 15
Cg2g020090	-1.83	Zinc finger CCCH domain-containing protein 18
Cg5g017170	1.85	Zinc finger CCCH domain-containing protein 20
Cg3g002440	-1.17	Zinc finger CCCH domain-containing protein 5
Cg4g005920	2.45	Zinc finger CCCH domain-containing protein 5
Cg2g003520	-1.42	Zinc finger CCCH domain-containing protein 56
Cg8g024210	1.09	Zinc finger CCCH domain-containing protein 66
Cg2g004190	-1.36	Magnesium-chelatase subunit ChlD, chloroplastic
Cg2g004200	6.04	Magnesium-chelatase subunit ChlD, chloroplastic
Cg9g021280	-3.68	Magnesium-chelatase subunit ChlH, chloroplastic
Cg2g043060	3.02	Magnesium-chelatase subunit ChlI, chloroplastic
Cg3g008610	-1.83	Calcium-transporting ATPase 12, plasma membrane-type

Cg3g010620	1.37	Calcium-transporting ATPase 12, plasma membrane-type
Cg1g006790	-1.35	Calcium-transporting ATPase 2, plasma membrane-type
Cg5g012700	-1.16	Calcium-transporting ATPase 9, plasma membrane-type
Cg6g017660	-3.85	Phosphate transporter PHO1 homolog 7
Cg6g025180	-1.03	Phosphate transporter PHO1 homolog 9
Cg7g015130	-1.11	Phosphate transporter PHO1 homolog 1
Cg7g005360	1.4	Metal tolerance protein 11
Cg9g029250	-2	Metal tolerance protein 10
Cg1g012170	-1.09	Metal tolerance protein 3
Cg2g022120	-2.46	Calcium-binding protein KRP1
Cg2g041040	-4.16	Calcium-binding protein KRP1
Cg2g022150	-1.71	Calcium-binding protein KRP1
Cg6g003330	2.02	Protein ALUMINUM SENSITIVE 3
Cg5g034040	4.37	Inorganic phosphate transporter 1-4
Cg5g035050	-3.68	Sulfate transporter 3.1

Table S9. Identification of DEGs related to transcription factor in leaves of *Citrus maxima 'Shatian Yu'* leaves under Aluminum stress

Gene ID	log2FoldChange	Description
Cg2g022720	-1.48	Transcription factor bHLH112
Cg9g001040	-1.45	Transcription factor bHLH147
Cg5g026370	1.79	Transcription factor bHLH153
Cg7g014890	-1.17	Transcription factor bHLH30
Cg9g022350	-1.48	Transcription factor bHLH49
Cg5g039930	-5.16	Transcription factor bHLH57
Cg8g007040	-2.49	Transcription factor bHLH63
Cg1g007400	3.73	Transcription factor bHLH93
Cg3g014510	-2.88	Transcription factor bHLH96
Cg4g018830	1.61	Transcription factor MYB1
Cg5g007970	1.43	Transcription factor MYB1
Cg7g021290	-5.11	Transcription factor MYB105
Cg5g038100	2.93	Transcription factor MYB12
Cg4g014320	1.08	Transcription factor MYB1R1
Cg9g010070	3.5	Transcription factor MYB2
Cg6g018200	1.6	Transcription factor MYB20
Cg2g012650	-4.73	Transcription factor MYB20
Cg5g002230	-1.83	Transcription factor MYB26
Cg5g040470	3.7	Transcription factor MYB3
Cg5g034000	2.45	Transcription factor MYB4
Cg2g003450	-3.05	Transcription factor MYB46
Cg8g017210	-1.98	Transcription factor MYB52
Cg3g017650	-1.05	Transcription factor MYB52
Cg5g039420	-2.02	Transcription factor MYB61
Cg1g009940	-6.89	Transcription factor MYB61
Cg5g022560	1.04	Transcription factor MYB62
Cg7g023280	-4.41	Transcription factor MYB83
Cg5g013190	-2.47	Transcription factor MYB86
Cg5g005500	-3.15	Transcription factor MYB86
Cg7g020880	-3.63	Transcription factor MYB88
Cg6g018450	4.49	Transcription factor MYB90
CgUng002190	-1.17	Transcription factor MYBS1
Cg9g002150	2.64	Ethylene-responsive transcription factor 1
Cg5g007750	2.57	Ethylene-responsive transcription factor 1
Cg5g044310	1.43	Ethylene-responsive transcription factor 13
Cg9g007940	5.07	Ethylene-responsive transcription factor 1B

Cg9g022790	2.98	Ethylene-responsive transcription factor 2
Cg2g042510	1.11	Ethylene-responsive transcription factor 3
Cg4g010160	1.35	Ethylene-responsive transcription factor 4
Cg9g022820	3.2	Ethylene-responsive transcription factor 5
Cg3g002850	1	Ethylene-responsive transcription factor CRF1
Cg4g007290	-4.23	Ethylene-responsive transcription factor ERF003
Cg1g019920	1.64	Ethylene-responsive transcription factor ERF003
Cg4g001690	-1.19	Ethylene-responsive transcription factor ERF003
Cg4g018510	2.69	Ethylene-responsive transcription factor ERF016
Cg4g017860	2	Ethylene-responsive transcription factor ERF017
Cg5g040600	-1.81	Ethylene-responsive transcription factor ERF023
Cg5g044100	-2.38	Ethylene-responsive transcription factor ERF034
Cg5g024830	1.71	Ethylene-responsive transcription factor ERF113
Cg3g021100	1.76	Ethylene-responsive transcription factor RAP2-1
Cg9g012290	-2.98	Ethylene-responsive transcription factor RAP2-4
Cg4g002330	-3.14	Ethylene-responsive transcription factor SHINE 2
Cg5g031000	-1.43	GATA transcription factor 1
Cg1g004300	-3.12	GATA transcription factor 18
Cg5g043880	-1.6	GATA transcription factor 2
Cg1g014090	-1.94	GATA transcription factor 2
Cg4g006690	-2.15	GATA transcription factor 21
Cg5g035280	-3.21	Transcription repressor OFP11
Cg1g023660	-1.9	Transcription repressor OFP17
Cg8g013110	-1.71	Transcription repressor OFP6
Cg6g014790	-4.04	Transcription repressor OFP6
Cg1g011490	1.33	Myb family transcription factor MOF1
Cg6g012570	2.89	Myb family transcription factor PHL5
Cg7g021270	-1.44	Myb family transcription factor PHL8
Cg7g023360	1.09	Nuclear transcription factor Y subunit A-1
Cg9g021740	2.33	Nuclear transcription factor Y subunit A-7

Table S10. Shatian pomelo leaf ratio of fresh weight/dry weight

Al concentration (mmol/L)	Leaf fresh weight (g)	Leaf dry weight (g)	Ratio=Leaf fresh weight/Leaf dry weight
0	0.76±0.052a	0.19±0.005a	3.92
1	0.60±0.028b	0.18±0.011b	3.42
2	0.46±0.026c	0.15±0.002c	3.12
4	0.43±0.030cd	0.13±0.007d	3.39
8	0.39±0.015d	0.14±0.005c	2.82

Table S11: Composition and content in hydroponic solution

Composition	Content (μmol/L)	Composition	Content (μmol/L)
NH4NO3	400	K2SO4	300
KNO3	1500	CuSO4·5H2O	0.5
MgCl2	25	ZnSO4·7H2O	1.5
Fe-EDTA (Na)	40	MnSO4·H2O	1.5
Ca(NO3)2·4H2O	1200	(NH4)5MoO24·4H2O	0.16
MgSO4·7H2O	500	KH2PO4	500
(NH4)2SO4	300	NaB4O7·10H2O	2.5

Table S12. Primers used for qPCR in the study

Gene ID	Gene name	Forward Primer (5' - 3')	Reverse Primer (5' - 3')
Cg6g023440	Gibberellin-regulated protein 14	GGTCAAACAAAGATTGCATCC	CTCTGTTGCCATAAGTTCCTG
CgUng005220	Auxin-responsive protein SAUR21	ACACTTAGCTGTCTATGTCGG	AACTCCTCTTCTGCCTGACTC
Cg2g044860	Gibberellin 2-beta-dioxygenase	CAAGAACGCTCACAGAAATTCC	CTGCCATCAATTCCATTACCTG

Cg6g023390	Peroxidase 63	CAATCACCAACAAGCAAATCAC	GAGGTTATGAGGATGGAGGAG
Cg1g021320	Metal transporter Nramp1	GTCTCTAGCAGCCAATCTCG	AGAAGAACACCAATCCACACTG
Cg1g014090	GATA transcription factor 2	CTAACTCCACTGATTCTTCCAC	AAAGCCATTCTAACTCTGCC
Cg9g020390	AP2-like ethylene-responsive transcription factor At2g41710	CCACTTCTGACCAACTATAACAC	TGCTCTCAGTCTTCGTAACC
Cg5g035050	Sulfate transporter 3.1	GTCGCCTCACTTCTCATAGC	GATAAACCCTAGCCTTAGCAGTC
Cg1g009400	Peroxidase 21	CACAGAATTGTCCGAAAGCAG	ATCACATGACTTGACGATGC
Cg4g018830	Transcription factor MYB1	CTTGGAAAGCCGTTGAAGAC	TCCCGCATCTCTTAAGACCT
Cg2g039090	β-tubulin	TGGATGTTGTTAGAAAGGAAGCTG	GATACCTTAGGAGACGGGAAGAC
